# Combating Medical Violence Against Deaf, DeafBlind, Blind and Partially Sighted Communities: A Community-Based Research Agenda for Canada and Abroad

**DOI:** 10.3390/healthcare14162446

**Published:** 2026-08-07

**Authors:** Sammy Jo Johnson, Yoonmee Han, Iffath Unissa Syed, Rachel da Silveira Gorman

**Affiliations:** 1 Department of Critical Disability Studies, York University, 4700 Keele Street, Toronto, ON M3J 1P3, Canada; sammyjo1@yorku.ca; 2 Department of Science and Technology Studies, York University, 4700 Keele Street, Toronto, ON M3J 1P3, Canada; yoom0303@yorku.ca; 3 Department of Health Policy and Administration, The Pennsylvania State University, 147 Shenango Avenue, Sharon, PA 16146, USA; 4 School of Health Policy and Management, York University, 4700 Keele Street, Toronto, ON M3J 1P3, Canada; gorman@yorku.ca

**Keywords:** Deaf, DeafBlind, blind, partially sighted, medical violence, healthcare, disability, ocularcentrism, audism, ableism

## Abstract

Introduction: Globally, disabled individuals experience persistent social inequalities and health inequities, yet the health and wellbeing of Deaf, DeafBlind, blind, and partially sighted (DDBBPS) people remain profoundly under-researched and excluded from social science and health policy agendas. Existing studies narrowly focus on narratives of hearing and vision impairments within a medical model of disability, which pathologizes difference and obscures the biomedical origins of social inequalities and health inequities experienced by these groups. Methods: Drawing on critical disability studies and community-based literature, this narrative review introduces and applies the concept of medical violence to examine how systemic ableism, audism, and ocularcentrism shape DDBBPS people’s healthcare experiences. Results: We identify six interrelated manifestations of medical violence: denied interpreting services, inaccessible health communication, harmful interpersonal practices, health inequities and medical avoidance, absence of DDBBPS practitioners, and gaps in community-based care. Conclusions: These conditions reinforce a cycle of exclusion and misrepresentation, wherein DDBBPS persons are denied equitable access to healthcare and are simultaneously constructed as objects of cure rather than as knowledge holders. We argue for a community-based participatory research agenda led by and for DDBBPS communities to challenge ableist research paradigms and advance health equity.

## 1. Introduction

In Canada, disabled individuals experience significant health disparities compared to non-disabled individuals, including higher rates of infection, severe outcomes, and death from the novel coronavirus (COVID-19) [1,2]. Yet, the health of disabled people remains at the margins of health equity research and agendas [3].

There is a notable lack of research on the health outcomes and care experiences of diverse Deaf, DeafBlind, blind, and partially sighted (DDBBPS) people in Canada. This gap persists even as many DDBBPS individuals experience overlapping systemic barriers—including ableism, classism, racism, and sexism—that reduce access to education, employment, livable income, housing, healthcare and other key social determinants of health. Existing health research suggests that DDBBPS communities experience higher rates of chronic conditions and worse health outcomes compared to sighted and non-Deaf individuals [4,5,6], and that the distribution of illness is disproportionately borne by individuals also impacted by racism, cisheterosexism, and other forms of systemic discrimination [7,8].

The negative health outcomes experienced by DDBBPS people are, in part, maintained by healthcare systems that are overwhelmingly inaccessible and habitually hostile. Nevertheless, when included in population health studies, there is a tendency to report narrowly on Deaf people’s hearing health [4] and blind and partially sighted (BPS) people’s vision health. A medical model of disability is pervasive in health research, wherein “disability is viewed as a deviation from the norm—an ‘undesirable difference’ that must be cured or repaired through medical and professional expertise” [9] (p. 369). Such common-sense assumptions are rooted in ideas of able-bodied, sighted, and hearing superiority and position disability as the research problem, delimiting “opportunities to study the social origins of disabled people’s health” [10], (para. 3). The result is a body of authoritative knowledge that is poorly equipped to address the everyday health concerns of diverse DDBBPS people, which often have less (or nothing) to do with vision/hearing and more (or everything) to do with the social and structural determinants of health inequities.

DDBBPS people’s health and healthcare experiences need to be gauged and researched outside of the narrow scope of medical hearing/vision health and access to hearing/vision screening and services, led by/for these communities. In particular, there is a need to better understand the ways in which ableism, ocularcentrism, and audism reduce access to care for diverse DDBBPS communities. Accordingly, we ask the following research question: how do ableism, audism, and ocularcentrism, among other forms of medical violence, impact DDBBPS people and limit access to care for these communities?

Structural barriers delimit access to care for DDBBPS communities in the context of medical violence. The DDBBPS acronym was developed as part of a larger project investigating the problems of medical bias and unmet health needs among racialized, disabled, women and trans people, and other equity-deserving groups [11]. The concept of medical violence is aligned with that of structural violence, the latter introduced and expanded by previous scholars [12,13]. The concept of structural violence affords “one way of describing social arrangements that put individuals and populations in harm’s way” [12] (p. 1686). Medical violence, on the other hand, focuses our attention on how the arrangement of medical institutions and the research bodies that support them put individuals in harm’s way.

Our purpose is to elucidate the overlapping forms of medical violence experienced by diverse DDBBPS persons and communities, which significantly reduces access to care for these communities and thus reproduces health inequities. We also aim to centre community perspectives, recognizing that much of the health research conducted on DDBBPS communities is not led by those with lived experience, a gap especially pronounced in Canada. To do so, we employ a narrative review of the literature, including both community-based and academic literature using a feminist [14] and situated search strategy. The rationale for this review is to generate knowledge in this area and recommend policies, practices, and guidelines to minimize harm to marginalized people and communities.

### 1.1. A Note on Terminology

We avoid terms such as ‘hearing impaired’ and ‘visually impaired,’ which construct deafness and blindness as medical deficiencies. We capitalize the terms ‘Deaf’ and ‘DeafBlind’ as part of a longer tradition used in and beyond Deaf studies, emphasizing that the Deaf community is a linguistic and cultural group. We recognize that there is no single experience of being Deaf and, similar to certain research and advocacy organizations [15], we use the term expansively to include those who identify as Hard of Hearing, DeafDisabled, and Late Deafened. Lastly, we use ‘blind and partially sighted’ to reflect the terminology used in the blind community and modes of writing used in the developing field of blind studies, led by BPS researchers.

We also use the acronym ‘DDBBPS.’ In doing so, we do not intend to conflate or collapse different lived experiences. Rather, drawing on the insights offered by disability justice organizers [16], we recognize the importance of cross-disability solidarity. Cross-disability solidarity involves resisting the ways in which disabled communities have been separated from each other and makes a commitment to ending all forms of disability oppression [16]. It necessitates an awareness of the many ways disabled lives are made not to matter outside of a medical model and the different ways disability oppression impacts our lives, recognizing that there is no single disability experience—an awareness that is needed if we are to work toward social change. Research that brings together these communities to examine how barriers show up in healthcare helps to clarify how different forms of disability oppression manifest in our institutions and the many policy and practice changes that are needed to promote access and equity [17].

### 1.2. Theoretical Framework: Medical Violence

We apply a framework of medical violence, which we conceptualize as the structural violence expressed through healthcare institutions, medical knowledge production, and medical professional guidelines, as they are currently organized in our capitalist social universe. Structural violence includes harm caused by social, political, economic, educational, and legal systems that perpetuate social inequalities and health inequities [18]. Structural violence impacts health and leads to diseases that are disproportionately correlated with low income, racialization, psycho-social/ chronic stress, and socioeconomic status disparities [19,20]. Medical violence, like the broader concept of structural violence, has material and ideological dimensions and consequences; it is differentially experienced according to social inequities based on race, class, gender, disability, sexuality, gender identity, and citizenship status [18,19,21]. Further, we conceptualize medical violence as encompassing negative medical interactions (NMIs), which can be articulated as oppressive interpersonal and systemic experiences at the point of receiving care; unmet health needs (UHNs), which include common ailments within a community and diminished access to care; and medically unexplained illnesses (MUIs), which refer to a range of symptoms not adequately explained or examined [11]. This framework is being developed as part of a larger research project that is led by the last author and co-led by the third author. The project aims to intervene in medical and artificial intelligence (AI) bias by creating an equity-based search algorithm that can be applied to electronic medical records, providing a means to track equity initiatives in healthcare [11].

Medical violence often converges with and results from related concepts of epistemic injustice and institutional ableism. Epistemic injustice is a harmful outcome against a marginalized group whereby individuals from the group, and/or the group as a whole, are discredited as sources of knowledge, expertise, and understanding. Epistemic injustice can be traced in institution-specific knowledge production, or society-wide in cultural ideologies [22]. In this sense, epistemic injustice as produced within medical institutions can result in medical violence when patient knowledge is discounted, and when culturally appropriate knowledge is absent. Importantly, epistemic injustice is often related to and based upon institutional ableism. Institutional ableism plays out against disabled populations when organizational policies, practices, and environments privilege non-disabled people and their lived knowledge and opinions while systematically disadvantaging disabled individuals [23]. In this sense, structural violence, epistemic injustice, and discrimination based on social locations of multiple marginalities are often interrelated and overlap with each other, and often manifest in the form of harmful and violent interactions and outcomes, within and through health policy, practice, and care settings, and research based on biomedical reductionism, which we define as medical violence.

However, in comparison to structural violence, epistemic injustice, and institutional ableism—concepts that can be used to highlight social injustice and harms in other social and political contexts—medical violence has a specific scope in policy and research as it refers to harm specifically inflicted through healthcare systems, clinical practices, or biomedical institutions [16]. This harm stems from traditionalist-oriented biomedical approaches, which are early perspectives that originated from the 19th century and continue to inform and dominate contemporary policy and are problematic because such perspectives define health as merely an absence of illness, disease, or condition, while anything that does not fit this definition is deemed to be abnormal, deficient, or deviant [21]. Medical violence is related to harm done within a medical setting and caused by health policies and practices based on the limited biomedical approach to health and illness. These violent practices include discrimination, lack of care and service, and differential treatment in health care systems based on social locations such as sex/gender, race, ethnicity, Indigenous/Aboriginal/Native status, (im)migrant status, class/socioeconomic status, age, religious beliefs, and ability/disability, among other things [19]. The violence results in physical, psychological, epistemic, and/or reproductive harm, and involves coercive, discriminatory, dehumanizing practices, omissions, or interventions caused within and through health care practices and interventions [24].

Building on this framework, we suggest that ableism, ocularcentrism, and audism are key forms of medical violence impacting DDBBPS communities. However, these concepts—especially ocularcentrism and audism—are not widely taken up in medical and healthcare research. Therefore, we outline these terms here:

Ableism: Population health literature seldom discusses medical ableism, defined, in part, as social devaluation that is experienced by people on account of their disabilities [25]. Ableism has also been defined as a system or network of norms, ideas, practices, and processes that produce a particular kind of body or embodiment that is projected as normal, perfect, or ideal while the parallel other is considered abnormal [23,26,27,28,29]. Within critical disability studies scholarship, it is explained that “ableism can lead to significant prejudice, and its ideas have been deeply embedded in conventional understanding of disability” [30] (p. 2).

Ocularcentrism: Ocularcentrism is a form of ableism, and specifically, a cultural belief in which blindness is rigidly constructed as the mere opposite of seeing [31,32]. This construction of blindness as a lack and an inferior way of being is embedded in everyday language, repeated in a variety of metaphors used in the dominant sighted culture [33]. The labelling and stigmatization of BPS people based on ocularcentric attitudes and perceptions restricts social participation and contributes to isolation [34].

Audism: In 1975, Deaf scholar Tom Humphries coined the term ‘audism,’ defining it as “the notion that one is superior based on one’s ability to hear or behave in the manner of one who hears” [35] (p. 240). The term describes both the systematic privileging of hearing modes of being in the world and the systematic disprivileging of Deaf modes of being. Such ideas permeate institutional practices in medicine, education, and family life focused on “disciplining Deaf bodies into becoming closer to normal hearing bodies” [35] (p. 245).

Importantly, ableism, ocularcentrism, and audism all intersect with other forms of oppression including racism, cisheterosexism, classism, and linguicism, which further reduce access to healthcare and relevant services for multiply oppressed disabled and DDBBPS persons. The knowledge that ableism is always shaped by other forms of privilege/oppression is central to the emerging disability justice movement [16].

The framework of medical violence informs our literature review, which sought to explore how these three aspects of medical violence—NMIs, UHNs, and MUIs—manifest in care interactions as experienced by DDBBPS people. We also employed this framework to critically examine how audism, ocularcentrism, and ableism saturate health care settings and shape the experiences of medical violence borne by DDBBPS communities.

## 2. Materials and Methods

This study adopts a purposive narrative review methodological framework and design [36,37] to examine key challenges and contexts shaping the aforementioned literature. “Key features of a narrative review include a flexible structure, a subjective approach to literature selection and interpretation, and a focus on providing a coherent narrative that integrates findings from different studies. Unlike systematic reviews, narrative reviews usually do not include a rigorous and reproducible methodology for literature search and selection. In terms of scope, a narrative review is broad and can include different types of studies, including qualitative and quantitative research, theoretical work, and expert opinion. Therefore, this type of review is useful for exploring new or complex topics, providing a comprehensive background and highlighting important themes and issues.” [38] (p. 4).

We used a theoretically informed purposive approach to sampling that did not aim to comprehensively or exhaustively map the existing literature. Rather, we aimed to explore a range of healthcare barriers impacting DDBBPS communities, incorporate the experiences of Deaf, DeafBlind, and BPS persons, and prioritize community conversations and knowledge production. Purposive sampling is a non-probability data collection technique in which researchers intentionally select sources because of their specific characteristics, experiences, or knowledge relevant to the research question [36,37]. As such, our method enabled critical engagement with literature, drawing on our professional and lived experience. We are a team of researchers from interdisciplinary fields, including: critical disability studies, science and technology studies, and health policy, management, and administration. The first author is a white, chronically ill researcher and Child of Deaf Adults (CODA) whose work focuses on Deaf community-engaged research in the GTA. The second author is a legally blind and racialized disability researcher who is involved in disability justice activism and advocacy for/by the BPS community, also in the GTA. The third author is a racialized feminist health researcher involved in advancing health equity of various vulnerable groups. The fourth author is a white-passing, mixed-race, sighted and hearing, disabled scholar.

### 2.1. Identification of Information Sources/Search Procedures—Databases, Organizations, Timeframe

Our narrative review [39] was conducted in three phases. In the first phase, we conducted a general search of Google Scholar © in May 2024. We used the following search phrase: “Deaf barriers to healthcare”. We limited the search to “Since 2020” to capture recent developments and contemporary policy/culture. As previously noted in other scholarly work using the same database, rather than conducting an exhaustive review of over 17,000 articles [40], we took screenshots of the first sixty articles and saved these results for our third phase (described below). The rationale for saving these screenshots was that retrieved results from Google Scholar ©, unlike academic databases, cannot be exported into .csv file types; search results are unique, change dynamically due to periodic updates, and the search cannot be replicated to yield the same articles even within a 24 h window. Also, we attempted to find journal articles by using the term “blind barriers to health care” and “DeafBlind barriers to health care”. However, the results were limited. The results included research that incorporated the word ’blind’ metaphorically to indicate research methods (e.g., double-blind or single-blind clinical research methods) and medical articles imbued with a reductive medical approach to blindness. As the result was saturated with a medical framework, we decided in our third phase to collect articles by using non-systematic and non-scoping review processes.

Our second phase involved a search using three academic databases: Web of Science, PubMed, and Medline (Ovid) in April 2026. Key terms used for the academic database search were “medical violence”, “blind”, “Deaf”, and “disability”, combined with Boolean operators (and/or), i.e., “(((medical violence) AND (Deaf)) AND (blind)) AND (disability)”. Although our database search terms may appear to be restrictive as stated above, the objective was not comprehensiveness but theoretically informed purposive sampling.

Timeframe: We limited our latter academic database search results to the past ten years (2015 to 2025) to capture a broader time period, e.g., pre and post- novel coronavirus (COVID-19) studies.

Geographic focus: We prioritized Canadian sources, but given our knowledge of the limited research devoted to this area, we selected international comparators when they offered relevant contribution to our frameworks.

Although database searches are an established mode of searching used in both systematic and non-systematic (narrative or scoping) literature reviews, they deprioritize community-based literature, such as reports and statements authored by community organizations, grassroots public awareness campaigns, and the like. It is also difficult when using database searches to prioritize research that is led by and/or inclusive of researchers with lived experience. This is only possible if and when authors situate themselves, often by incorporating a reflexivity or positionality statement. Though increasingly commonplace in health equity scholarship, many researchers still do not situate themselves in their writing [41]. At the same time, disability-related research has historically been dominated by non-disabled researchers [41]. In response, we implemented a third phase of searching that allowed us to prioritize community-based knowledge generation and academic research led by and/or inclusive of DDBBPS researchers.

The third phase of our search employed a decidedly feminist approach [14]. In this phase, we combined and incorporated both scholarly and grey literature, drawing on our lived experience and social locations—particularly authors one and two—to conduct our search. More specifically, we started this phase by searching where we were familiar with literature, including the lists from the literature identified in the first phase and emerging community conversations we were familiar with from our social locations as one CODA and one BPS researcher. This step involved searching for articles associated with the Deaf Hub located at the National Technical Institute for the Deaf (NTID). The Deaf Hub is part of a pipeline of Deaf scientists and healthcare professionals that has been disassembled under the current attack on equity, diversity, and inclusion in the US [42], highlighting the importance of our call for more community-led research. Searching here was a purposeful strategy to incorporate research projects inclusive of and/or led by Deaf researchers. We also searched the websites of community organizations we were familiar with to identify reports and other publications related to healthcare access for DDBBPS communities. This phase also involved forward and backward citation searching to identify other relevant sources. Grey literature included panels, presentations, and reports published by community-based organizations.

We have not included a Preferred Reporting Items for Systematic reviews and Meta-Analyses (PRISMA) statement, as it would be inappropriate for narrative reviews with purposive sampling and is more aligned with systematic reviews or meta-analyses [43,44]. “Narrative literature review articles are publications that describe and discuss the state of the science of a specific topic or theme from a theoretical and contextual point of view.” [45] (p. 1). Indeed, several narrative reviews and narrative syntheses conducted and published previously do not utilize PRISMA [46,47,48,49,50,51,52].

### 2.2. Screening, Eligibility, Inclusion/Exclusion, and Data Analysis

Four reviewers screened Google Scholar © records together, while one reviewer independently screened academic database sources. Three reviewers reviewed the grey literature. Any disagreements were addressed through discussion in team meetings until consensus was reached. We screened titles of journals, monographs, and books, and did a secondary screening of abstracts. We then proceeded with a full review of sources that met our inclusion criteria. Our inclusion/exclusion criteria were as follows:

(i) We included full-text journals, dissertations, chapters in books, conference proceedings, and community-based outputs.
(ii) We included sources that focused explicitly on the healthcare experiences of DDBBPS people and communities.
(iii) We excluded sources that were not available in the English language or American Sign Language (ASL).
(iv) We excluded sources that did not focus on the first-hand experiences of DDBBPS individuals and/or focused on other institutional settings (e.g., education) rather than healthcare. For instance, exclude: ASL interpreters’ perceptions of communication barriers (for Deaf and Hard of Hearing (HOH patients)) as opposed to Deaf and HOH patients’ perceptions of barriers to communication.
(v) We excluded sources with explicit medicalized frameworks that focused on deafness, blindness, and/or deafblindness as medical problems to be cured. For instance, we excluded papers examining access to infant hearing screening programs.

Data were analyzed thematically using manual open coding by two researchers. Thematic saturation was achieved when no new insights emerged. All included sources were analyzed by two researchers, with the exception of ASL sources, which were only reviewed by the first author (who is fluent in ASL). In line with our feminist approach, we did not approach coding as a neutral process and our focus was not on creating or ensuring consistent codes between coders. Rather, we implemented a reflexive approach [53], drawing on our expertise as one CODA and one partially sighted scholar. A feminist, reflexive approach to coding can enhance reliability by allowing researchers to draw on our unique social locations as resources [53]. Using this approach, the second coder focused on identifying any gaps or disagreements, which enabled us to engage more thoroughly with the sources. As researcher-stakeholders, we examined our own feelings, motives, and biases to understand how they influenced our thoughts and actions and discussed these, as well as coding discrepancies and differences, in team meetings [54].

## 3. Results

**Figure 1:**
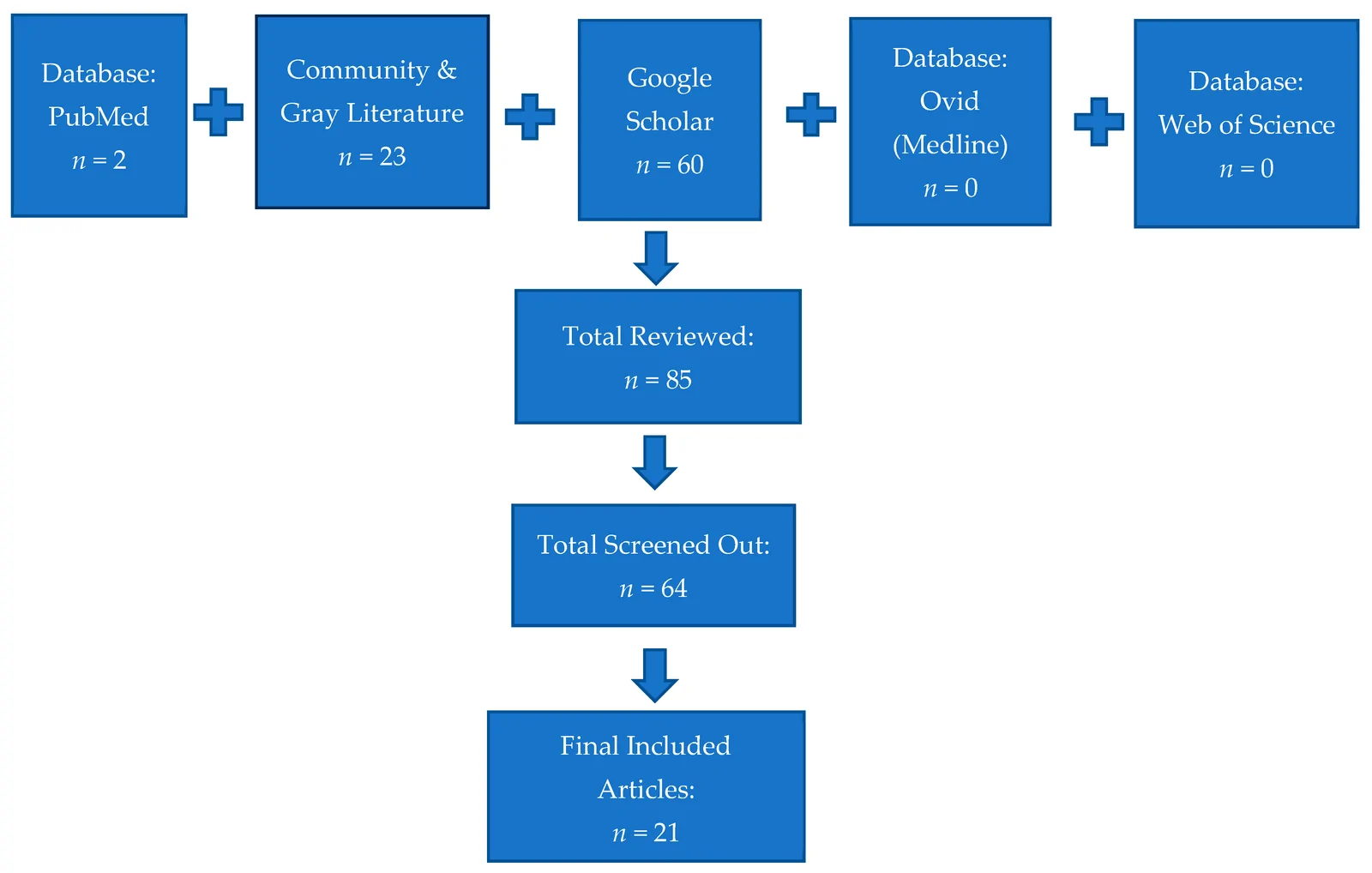
Flow Chart of Search Strategy. Our academic database search resulted in two articles. Our search of the sixty Google Scholar © sources yielded four articles, while our search of the grey literature yielded 23 sources, of which six were screened out. A total of 21 sources were included in the final set and thematically analyzed.

Our search strategy is demonstrated in a flow chart in Figure 1 indicating that eighty-five total sources were screened and analyzed from PubMed (*n* = 0 out of 2 included), Google Scholar (*n* = 4 out of 60 included), and grey literature (*n* = 17 out of 23 included). *n* = 21 sources were included in our final thematic analysis [55] and organized in Table 1. These sources comprise the following:

- Peer-reviewed journal articles: *n* = 15 (71%)
- National or professional organizations, policy guides, reports, or other publications: *n* = 6 (29%)

As indicated, very few peer-reviewed articles exist that document medical violence on Deaf, blind, and DeafBlind people, including those with disabilities in Canada (14%, *n* = 3), although there is a growing body of research focusing on the healthcare experiences of Deaf persons in the US (62%, *n* = 13) and abroad (23%, *n* = 5). While it was our intention to uncover how the three aspects of medical violence—namely, NMIs, UHNs, and MUIs—our review did not uncover MUIs experienced by DDBBPS persons. This outcome is explained by the lack of research with these communities.

## 4. Negative Medical Interactions

### 4.1. Interpreting Denied

Studies from the US show that it is not uncommon for Deaf individuals’ requests for sign language interpretation to be denied or ignored in healthcare settings [56,57,58,59]. Despite decades-long legal protections confirming the right to sign language interpreting in hospitals and other healthcare settings in Canada, requests for interpretation regularly go unmet [74]. In a recent qualitative study in Ontario, it was found that Deaf participants were frequently denied access to ASL interpreters “because hospitals refused to book them, indicating that they are costly, and leaving patients to pay for interpreters themselves” [17] (p. 4). Research in the US suggests that low-income individuals are disproportionately impacted, particularly as public clinics may be deterred from booking interpreters due to concerns about demands on time and anticipated costs and lack of familiarity with reimbursement mechanisms [56]. Barriers to communication and care include not only a persistent failure to meet interpreting requests, but issues around qualifications [74] and quality, particularly when using telehealth and video remote interpreting [58,59]. Research in the US suggests that Deaf patients may have to undertake even more labour to arrange interpreting for virtual visits due in part to technological barriers preventing three-way video between patient, provider, and interpreter [58].

Without access to a qualified interpreter, Deaf and DeafBlind individuals may face significant barriers when explaining their health concerns to non-Deaf, non-DeafBlind, and non-signing practitioners and receiving information about their own care. This puts Deaf and DeafBlind individuals at increased risk of miscommunication and misinformation [59,60,61,62,63]. Such risks are unavoidable given the current shortage of qualified ASL and Langue des Signes Québécoise (LSQ) interpreters in Canada—sharply felt in rural areas [74]—as well as DeafBlind interpreters and intervenor services [64,75]. Also noted in the US-based literature, there is a need for greater numbers of Black and racialized interpreters and interpreters skilled in Black American Sign Language and non-ASL signed languages; without such representation, the above communication barriers are exacerbated for racialized and linguistically diverse community members [7,60,61,65].

### 4.2. Lack of Access to Health Information and Communication

Existing research also indicates that health information related to one’s diagnosis, treatment plans, medications, healthcare services, facilities and equipment is consistently delivered to BPS and DeafBlind individuals using inaccessible formats [17,64,76,77]. The reliance on written and visual communication in healthcare settings to convey information about general health (e.g., pamphlets and videos) and one’s own care (e.g., image-based tests and medication information), as well as initiate care (e.g., registration forms and appointment letters) is routinized in care provision—at the same time, practitioners and staff may be unsure how to provide accessible options or unwilling to do so [17,76,77,78]. Studies in the UK and US have shown that DDBBPS people rely on family and friends to navigate inaccessible healthcare settings [59,76,77]. This not only creates a precarious kind of access, wherein information about one’s own care or even the ability to initiate care is dependent on assistance that can be denied or withdrawn, but it also frequently leaves DDBBPS persons with a diminished level of care compared to sighted and hearing persons. Moreover, such individual ‘solutions’ fail to address systemic barriers including inaccessible design and physical barriers such as insufficient lighting and signage reported by DeafBlind persons [66], advocated for by organizations serving BPS communities [67], and the lack of clear policies and protocols to respond to interpreting and other access requests [17].

For DDBBPS people, barriers to communication and information in hospitals can result in additional NMIs, including misdiagnosis, medication errors, delays in care, coercive care experiences, and avoidance due to the fatigue and trauma that comes with navigating hostile settings [17,60,61,62,64,67]. Furthermore, studies in the US and UK indicate that information and communication barriers are compounded for BPS community members without class privilege, including those who cannot afford up-to-date technology and those less familiar with navigating healthcare systems [76,77]. In a recent qualitative study in Columbia, researchers found that participants with lower income and lower educational levels experienced heightened barriers when accessing specialized care [68].

### 4.3. Harmful Interpersonal Practices

According to researchers, audism can manifest as the “dismissal of needs by refusing to provide interpreters or in-depth health information” [57] (p. 519). In Ontario, healthcare providers “may not be incentivized to spend extra time to fulfill patients’ communication needs because they are often remunerated only for visits of a particular length (e.g., 10–15 min)” [17] (p. 11). This focus on profit and speed disproportionately harms racialized Deaf individuals, especially when interpretation requires additional time or involves more than two languages—for instance, when patients and families use a signed or spoken language other than ASL or English, or when individuals are unfamiliar with the medical system [60]. The routine failure to provide accessible information and communication perpetuates hearing/sighted dominance in healthcare settings as DDBBPS persons are expected and required to fit into a sighted, hearing, and able norm. Not only are power imbalances rooted in notions of sighted/hearing superiority that inform how patients are or are not allowed to take up space and communicate, but they are complicated by a patient-provider dynamic. The literature is clear that it is profoundly disempowering to have one’s access needs repeatedly sidelined; yet the violence of this act can be obscured when providers assume that they know best. For example, sighted practitioners may assume that large print is sufficient for accessing visual health information for all BPS people [76]. These kinds of assumptions also show up when Deaf patients’ requests for on-site interpreters are ‘met’ with video remote interpreting (VRI). As found in one Florida-based study, this frequently happened to Deaf patients even when it was explicitly stated that they would have preferred to use “‘anything but VRI’” [59] (p. 53), due to frequent connectivity issues among others that significantly reduced the quality of care received.

When providers assume that they know best or that patients can make do without requested access services, an unequal standard of care experienced by DDBBPS persons becomes normalized. As indicated in the aforementioned research [59] in reference to the reliance on VRI services, despite the self-advocacy patients put in to fight for on-site interpreting, such maneuvers can provide the appearance of accessibility for providers that is not actually experienced by patients.

Lastly, audist and ocularcentric attitudes in healthcare may result in the deprioritization of DDBBPS patients’ health concerns. This can involve a preoccupation with disability at the expense of the actual health concerns of patients. For example, in one study, a participant “shared that after they experienced the loss of their third child, a provider seemed more preoccupied with the fact that they had braille grief resources to share rather than offering comfort” [17] (p. 10). It can also involve what is called “practitioner misconception” [69] (p. e167), wherein practitioners’ internalized ideas that communication with Deaf patients is too challenging bias practitioners against providing particular forms of routine care, thus driving UHNs. Practitioner misconception “may create a higher threshold in an ED practitioner’s mind prior to deeming a procedure necessary, resulting in lower rates of routine types of care to Deaf individuals in the ED setting compared with patients who communicate primarily in English” [69] (p. e168). Further research is needed to examine the interaction between racism and sexism that inform whose pain is believed as well as audism and ocularcentrism, particularly as research already indicates that linguistic and racial discordance between patients, providers, and interpreters can contribute to the dismissal of Black Deaf women’s concerns [70].

## 5. Unmet Health and Social Care Needs

### 5.1. Health Inequities and Medical Avoidance: A Vicious Cycle

Unmet health needs include the higher rates of chronic conditions mentioned at the start of this paper. Unmet social care needs are also important and impact health and wellbeing. For example, a study in Norway found that loneliness was a significant issue for BPS people, although the term visually impaired is used more explicitly [71]. More broadly, community-based reporting indicates that “under-diagnosis and under-treatment of potentially serious conditions is more common in Deaf people” [79] (p. 3). While there is less research on BPS and DeafBlind persons’ health outcomes, similar inequities persist due to unequal treatment in care and disparities in access to the social determinants of health. For instance, BPS people experience high rates of unemployment and are thus subject to material deprivation and poorer health outcomes.

Moreover, NMIs are drivers of medical avoidance. Differently put, ocularcentrism, audism, ableism, racism, cisheterosexism, classism, and other overlapping forms of oppression lead to medical avoidance; most impacted are those harmed by more than one oppressive structure (e.g., audism and racism) [60]. In addition, when access remains uncertain, the burden of navigating care often falls to DDBBPS individuals. Yet, the repeated experience of trying to negotiate access not only causes fatigue and exhaustion [62], but it also increases the likelihood that DDBBPS people will not receive timely treatment, particularly as individuals may avoid medical care due to systemic oppression. For instance, audist cisheterosexism in medical systems drives medical avoidance and contributes to under-diagnosis and adverse outcomes related to delayed diagnosis and treatment among Deaf LGBTQ populations [8].

Additionally, unequal access to the social determinants of health means that even reaching a hospital or other medical setting can be a barrier, just as the costs associated with care can contribute to avoidance. In a Toronto-based qualitative study, DeafBlind participants described the inadequacies of subsidized dental care under the Ontario Disability Support Program and reported avoiding care due to anticipated costs [80]. These experiences highlight the way that poverty reinforces exclusion and oppression [81], and the need to go beyond attitudinal changes in order to combat ableist, audist, and ocularcentrist inequities in healthcare access.

### 5.2. Absence of DDBBPS Practitioners

The scarcity of DDBBPS care providers also drives a lack of access to care. Research in the US shows that Deaf women may travel significant distances to access a provider with experience supporting Deaf patients [57]. This lack of access to signing fluent providers and those with shared lived experience is further exacerbated for negatively gendered and racialized persons [70]. In a recent US study, researchers note that, at the time of publication, “there are no known Black DHH [Deaf or Hard of Hearing] physicians that use ASL” [7] (p. 2). The total exclusion of Black Deaf ASL users from physician roles is an outcome of audism and racism entrenched in education systems [61]. There is less research examining the care experiences of BPS and DeafBlind persons. Further research should ask: Where do BPS and DeafBlind individuals go to access care and why? What difference does matched lived experience make? And how can we address barriers these communities face in entering healthcare professions?

### 5.3. Gaps in Community-Based Care

DDBBPS people also experience a lack of access to community-based care. This means that for some individuals, there can be few options outside the hospital to receive care. For instance, Deaf individuals may be more likely to use EDs compared to non-Deaf individuals due to a lack of access, including interpreters in other settings [72]. This goes beyond medical avoidance and can be more accurately described as medical exclusion. In addition, the lack of accessible options in the community can lead to prolonged stays in hospital and psychiatric institutions [62], and are thus causes of NMIs (i.e., institutionalization).

Lack of access to community care also involves access to community-based health education and information sessions, which can intensify information privation already disproportionately impacting these communities. These educational spaces may rely heavily on both visual formats and spoken language (e.g., presentations, slides, and video), with requests for access (e.g., sign language interpretation, closed captioning, visual descriptions, plain language, and/or alternative digital options) frequently denied [17]. The lack of accessible health-related information can limit the possibilities for DDBBPS people to be informed about their own health and make decisions about their own care [17,73,78], as well as perpetuate unequal access to health literacy resources.

## 6. Discussion—Toward a Community-Based Research Agenda

Our review confirms the lack of academic research that takes the health/care experiences of DDBBPS communities into account while highlighting the urgent need to address this gap. Indeed, our database search revealed only four studies that met our inclusion/exclusion criteria. While there is a growing body of international academic research, mostly US-based, which examines barriers to care experienced by Deaf individuals [56,57,58,59,69,70], there is considerably less research that explores the experiences of DeafBlind and BPS individuals at the point of receiving care. This imbalance in the evidence base is reflected in our review: the majority of sources (*n* = 14, 67%) focus on the health and care experiences of Deaf individuals and communities, while BPS and DeafBlind experiences are underrepresented in the data. One additional source [17] explores the experiences of both Deaf and BPS individuals. While some of these articles include the experiences of DeafBlind individuals, most do not. Ultimately, research on disability communities continues to be shaped by medical model frameworks [81], which not only limits our awareness and understanding of the barriers to care experienced by diverse disability communities, but it also reinforces a preoccupation with deafness and blindness as medical problems to be ‘fixed.’ Our review emphasizes the need to put ableism, ocularcentrism, and audism—as institutional problems requiring immediate attention—on the research agenda.

Our study also reveals that DDBBPS communities are impacted by various and overlapping forms of medical violence that reduce access to care and perpetuate health inequities. More specifically, our findings show that DDBBPS individuals frequently experience barriers to communication and information about their health, including a lack of interpreters. These NMIs drive miscommunication, misinformation, and coercive care. NMIs also include overt audism and ocularcentrism—such as refusal of access requests, care denial, and minimization of health concerns—which reinforce medical avoidance. Additional UHNs include the absence of DDBBPS practitioners and community-based health education.

Intervening in medical violence requires more than learning terms like audism and ocularcentrism. Future research must make a serious attempt to examine how and where ableism, ocularcentrism, audism, and other forms of violence manifest in health care settings. We call for politically guided research [82] grounded in the lived experiences of DDBBPS communities—research **by and for**, not **on**, these communities. As previously noted, disability populations have long been used as research objects rather than beneficiaries [83].

Future research is needed that explores the healthcare experiences of DDBBPS communities, particularly within the Canadian context. Inequitable access to communication and information—including information about one’s own health, diagnosis, medication, and so on—is a life-and-death issue. It can take a lot of work to navigate inaccessible healthcare settings, something our review helps to highlight. This labour often comes at a cost, including fatigue, burnout, and exhaustion. There is a need for future research to take on some of the work of improving accessibility in our healthcare institutions in collaboration with DDBBPS communities, such as co-designing and co-developing institutional policies and processes aimed at improving access to communication and information in healthcare settings, as well as access to community-based care.

Research on DDBBPS people remains dominated by cure-oriented agendas aiming to eradicate deafness and blindness, while equity-focused studies are scarce in Canada. Our findings, however, highlight the structural and institutional barriers that limit access to care for DDBBPS communities, including a lack of access to interpreters, routine exposure to audist, ocularcentrist, and other discriminatory practices in healthcare, medical avoidance, a lack of DDBBPS practitioners, and gaps in community-based care. These, we argue, must be urgently incorporated into the health equity research agenda along with a commitment to the principles of community-based research. As critical disability scholars have noted, “community-based research principles have the power to confront and transform institutionally entrenched forms of disability research” [83] (p. 11), and to make sure that communities directly benefit from an expanded research agenda.

## 7. Conclusions

In this paper, we have conducted a narrative review of the literature to generate knowledge about the health/care experiences of DDBBPS communities. We have discussed the structural barriers that limit access to care for DDBBPS communities in the context of medical violence. Drawing on critical disability studies, blind studies, and Deaf studies scholarship, we also outlined and discussed three oppressive systems—ableism, ocularcentrism, and audism—the latter two likely new to many readers. Our work affirms there is a notable lack of research on the health outcomes and care experiences of diverse DDBBPS people.

One of the strengths of our work is that it contributes to a better understanding of how audism and ocularcentrism impact diverse DDBBPS communities’ access to care, as described in both community-based and academic literature. Another strength is that our work is informed by a growing body of work on Deaf health in the US, led by and/or inclusive of Deaf researchers. Those at the forefront of this work include researchers at the Rochester Institute of Technology/National Technical Institute of the Deaf and the Deaf Hub.

However, our work is not without limitations. One of the limitations of our work includes the relatively limited empirical base identified through the structured database review, and the small number of empirically based peer-reviewed studies which met the inclusion criteria. Accordingly, part of our evidence comes from grey literature, reports, videos, and community sources, based largely on the authors’ firsthand knowledge. We also acknowledge that our review attempted to combine very diverse populations (Deaf, DeafBlind, blind, and partially sighted individuals) whose experiences, identities, and healthcare needs may differ substantially. Combining different disability groups risks flattening different experiences. To reduce this risk, we have tried to remain attentive to the different ways in which audism and ocularcentrism manifest in healthcare settings and interactions, as well as where and how these forms of oppression overlap. Typically, research focuses on either Deaf or BPS communities, often leaving DeafBlind communities out of the conversation. By bringing the experiences of Deaf, DeafBlind, and BPS communities together, our discussion emphasizes that there is no single experience of disability oppression and that there is no single fix or solution. Another limitation is that although our search strategy used multiple databases, it was not exhaustive.

Our work also points to some areas for future research and carries implications for healthcare practice, professional education, and policy. One important recommendation is to include relevant concepts, terminology, and frameworks in healthcare professional education, including social and cultural models of disability and Deaf identity. This raises important questions about who is qualified to deliver this kind of education and training, and highlights the need to work with DDBBPS communities to design and provide professional education related to anti-ableism, access, and disability justice. Recognizing that DDBBPS and other disability communities hold the knowledge and expertise necessary to lead this work challenges the hierarchical ordering of knowledge that privileges medical expertise over community and experiential knowledge, which is a form of epistemic injustice.

There are also opportunities to improve accessibility standards, especially for service organizations, and to enhance policy development. For example, there is an urgent need for community co-designed policies aimed at ensuring that requests for access are meaningfully responded to by healthcare practitioners and institutions, including policies related to booking and providing interpreters. Such policies should respect the preferences of patients, including preferences for specific interpreters and in-person rather than virtual interpreting services. Practitioners also need to be supported to develop the knowledge needed to meaningfully and promptly respond to access requests. Yet access cannot be dependent on the goodwill of individual practitioners; rather, it requires systemic interventions informed and guided by community expertise.

Lastly, our study emphasizes the need for a community-based research agenda and to move beyond a medical model in health equity research. Future research must also go beyond “inclusion” framed as awareness training, which fails to address structural oppression. Instead, it must examine how audism and ocularcentrism limit access to education, employment, housing, income, and other social determinants of health. We argue that both should be explicitly recognized as determinants of health inequities and that DDBBPS communities’ knowledge must guide efforts to challenge medical violence and achieve equitable care in Canada.

## Figures and Tables

**Table 1:** Results of Thematic Analysis from the Literature Review. A total of 21 articles were thematically analyzed and organized by author, year, source type, characteristics -theme/main findings, and country or region.

No.	Author	Year	Source Type	Characteristics-Theme/Main Findings	Group	Country	Reference Citation
1	Perrodin-Njoku et al.	2022	Journal	Unmet health and social care needs; medical avoidance; racism; lack of access to Black Deaf and racialized Deaf care providers and Black and racialized interpreters	Deaf/HOH	United States	[7]
2	Kushalnagar & Miller	2019	Journal	Unmet health and social care needs; medical avoidance; audism; cisheterosexism; negative medical interactions	Deaf	United States	[8]
3	Saeed et al.	2022	Journal	Negative medical interactions; audism; ocularcentrism; dismissal of health concerns; lack of access to communication and information needs in prenatal care services	Sensory, intellectual, developmental disabilities	Canada	[17]
4	Schniedewind et al.	2020	Journal	Negative medical interactions; unmet interpreting requests; lack of access to qualified interpreters	Deaf	United States	[56]
5	Panko et al.	2023	Journal	Negative medical interactions; lack of access to communication; lack of access to perinatal information; unmet health and social care needs; audism	Deaf	United States	[57]
6	Mussallem et al.	2022	Journal	Negative medical interactions; diminished access using VRI; increased burden of coordinating access	Deaf	United States	[58]
7	James et al.	2022	Journal	Negative medical interactions; unmet interpreting requests; lack of access to information; diminished access using VRI; audism	Deaf	United States	[59]
8	National Association of the Deaf	2023	Non-Profit Civil Rights Organization	Unmet health and social care needs; lack of access to information; diminished access using VRI; intersectionality; barriers to care compounded for racialized and low-income individuals; medical avoidance; audism; racism	Deaf	United States	[60]
9	Michigan Medicine	2022	Academic Medical Center affiliated with University of Michigan	Negative medical interactions; audism; racism; unmet health and social care needs; unmet interpreting requests; lack of access to culturally relevant care; lack of access to Black Deaf and racialized Deaf care providers and Black and racialized interpreters; medical avoidance	Deaf	United States	[61]
10	NHS Deaf Mental Health Working Group	2023	Collaborative Partnership with NHS England and SignHealth	Negative medical interactions; audism; lack of access to interpreters; compounded barriers; unmet health and social care needs; increased burden of coordinating access; barriers to community-based care; medical avoidance	Deaf	United Kingdom	[62]
11	Lim et al.	2025	Journal	Negative medical interactions; communication barriers in pharmacy services	Deaf, DeafBlind, HOH	Canada	[63]
12	Jaiswal et al.	2020	International Non-Profit Organization, Magazine of Deafblind International	Negative medical interactions; lack of access to information; gaps in intervenor services	DeafBlind	Canada	[64]
13	Franklin	2021	Local News for Rochester, NY	Negative medical interactions; intersectionality, lack of access to Black Deaf and racialized Deaf care providers; lack of access to culturally relevant care	Deaf	United States	[65]
14	Fernández-Valderas et al.	2017	Journal	Negative medical interactions; lack of access to information and communication; inaccessible environments; audism; ocularcentrism	DeafBlind	Spain	[66]
15	Blind Citizens of Australia	2020	National Organization	Negative medical interactions; lack of access to information; inaccessible physical environments; addressing ocularcentrism	Blind	Australia	[67]
16	Oviedo-Cáceres et al.	2023	Journal	Negative medical interactions; lack of access to information; inaccessible environments; compounded barriers for individuals with less access to resources and support; intersectionality	Low-vision (Partially sighted)	Colombia	[68]
17	Conner et al.	2023	Journal	Negative medical interactions; lack of communication access in emergency care services; audism; dismissal of health concerns	Deaf	United States	[69]
18	Helm et al.	2023	Journal	Unmet health and social care needs; negative medical interactions; audism; lack of communication; lack of access to information; lack of access to Black Deaf and racialized Deaf care providers and Black and racialized interpreters; intersectionality	Deaf/HOH	United States	[70]
19	Brunes et al.	2019	Journal	Unmet health and social care needs; social isolation and loneliness	Visually impaired/ blind/ partially sighted	Norway	[71]
20	McKee et al.	2015	Journal	Unmet health and social care needs; lack of access to interpreting	Deaf	United States	[72]
21	Paracha et al.	2023	Journal	Negative medical interactions; lack of ASL interpretation in pharmacy services	Deaf	United States	[73]

## Data Availability

The raw data supporting the conclusions of this article will be made available by the authors on request.

## References

[1] Brown H.K., Saha S., Chan T.C.Y., Cheung A.M., Fralick M., Ghassemi M., Herridge M., Kwan J., Rawal S., Rosella L. (2022). Outcomes in patients with and without disability admitted to hospital with COVID-19: A retrospective cohort study. Can. Med. Assoc. J..

[2] Lunsky Y., Durbin A., Balogh R., Lin E., Palma L., Plumptre L. (2022). COVID-19 positivity rates, hospitalizations and mortality of adults with and without intellectual and developmental disabilities in Ontario, Canada. Disabil. Health J..

[3] Downer M.B., Rotenberg S. (2023). Disability—A chronic omission in health equity that must be central to Canada’s post-pandemic recovery. Health Promot. Chronic Dis. Prev. Can..

[4] Woodcock K., Pole J.D. (2007). Health profile of deaf Canadians: Analysis of the Canada Community Health Survey. Can. Fam. Physician Med. Fam. Can..

[5] Crews J.E., Chou C.-F., Sekar S., Saaddine J.B. (2017). The Prevalence of Chronic Conditions and Poor Health Among People With and Without Vision Impairment, Aged ≥ 65 Years, 2010–2014. Am. J. Ophthalmol..

[6] Rotenberg S., Davey C., Banks L.M. (2025). Health, education and well-being for children with deafblindness: A secondary analysis of 36 Multiple Indicator Cluster Surveys. Arch. Dis. Child..

[7] Perrodin-Njoku E., Corbett C., Moges-Riedel R., Simms L., Kushalnagar P. (2022). Health disparities among Black deaf and hard of hearing Americans as compared to Black hearing Americans: A descriptive cross-sectional study. Medicine.

[8] Kushalnagar P., Miller C.A. (2019). Health Disparities Among Mid-to-Older Deaf LGBTQ Adults Compared with Mid-to-Older Deaf Non-LGBTQ Adults in the United States. Health Equity.

[9] Hayes J., Hannold E. (2007). The Road to Empowerment: A Historical Perspective on the Medicalization of Disability. J. Health Hum. Serv. Adm..

[10] Mannor K.M., Needham B.L. (2024). The study of ableism in population health: A critical review. Front. Public Health.

[11] da Silveira Gorman R., Syed I.U., El Morr C., De-Lawrence L., Dinca-Panaitescu S., Butler A., Muhlenbach F., Maret P., Berthelot-Raffard A., Mgwigwi T. (2023). Training an AI to Detect Medical Bias and Unmet Health Needs Through Critical Race and Disability Theory and Community-Generated Data.

[12] Galtung J. (1969). Violence, Peace, and Peace Research. J. Peace Res..

[13] Farmer P.E., Nizeye B., Stulac S., Keshavjee S. (2006). Structural Violence and Clinical Medicine. PLoS Med..

[14] Syed I.U. (2021). Feminist political economy of health: Current perspectives and future directions. Healthcare.

[15] (2026). National Deaf Center on Postsecondary Outcomes. Deaf Awareness. https://nationaldeafcenter.org/resources/deaf-awareness/.

[16] Sins Invalid (2019). Skin, Tooth, and Bone: The Basis of Movement Is Our People.

[17] Saeed G., Brown H.K., Lunsky Y., Welsh K., Proulx L., Havercamp S., Tarasoff L.A. (2022). Barriers to and facilitators of effective communication in perinatal care: A qualitative study of the experiences of birthing people with sensory, intellectual, and/or developmental disabilities. BMC Pregnancy Childbirth.

[18] Syed I.U.B. (2015). Labour Exploitation and Health Inequities among Market Migrants: A Political Economy Perspective. J. Int. Migr. Integr..

[19] Syed I.U. (2020). Racism, racialization and health equity in Canadian residential long term care: A case study in Toronto. Soc. Sci. Med..

[20] Burton C.W., Gilpin C., Draughon Moret J. (2021). Structural violence: A concept analysis to inform nursing science and practice. Nurs. Forum.

[21] Syed I.U. (2019). In Biomedicine, Thin Is Still In: Obesity Surveillance among Racialized, (Im)migrant, and Female Bodies. Societies.

[22] Fricker M. (2007). Epistemic Injustice: Power and the Ethics of Knowing.

[23] Campbell F.K. (2009). Contours of Ableism.

[24] Metzl J.M., Hansen H. (2014). Structural competency: Theorizing a new medical engagement with stigma and inequality. Soc. Sci. Med..

[25] Janz H.L. (2019). Ableism: The undiagnosed malady afflicting medicine. Can. Med. Assoc. J..

[26] Campbell F.A. (2001). Inciting legal fictions: Disability’s date with ontology and the ableist body of the law. Griffith Law Rev..

[27] Jain N.R. (2022). The capability imperative: Theorizing ableism in medical education. Soc. Sci. Med..

[28] Reynolds J.M. (2018). Three Things Clinicians Should Know About Disability. AMA J. Ethics.

[29] Wendell S. (2001). Unhealthy Disabled: Treating Chronic Illnesses as Disabilities. Hypatia.

[30] Borowsky H., Morinis L., Garg M. (2021). Disability and Ableism in Medicine: A Curriculum for Medical Students. MedEdPORTAL.

[31] Bolt D. (2014). The Metanarrative of Blindness: A Re-Reading of Twentieth-Century Anglophone Writing.

[32] Bolt D. (2023). Disability Duplicity and the Formative Cultural Identity Politics of Generation X.

[33] Rodas J.M. (2009). On Blindness. J. Lit. Cult. Disabil. Stud..

[34] Bulk L.Y., Smith A., Nimmon L., Jarus T. (2020). A closer look at opportunities for blind adults: Impacts of stigmatization and ocularcentrism. Br. J. Vis. Impair..

[35] Bauman H.-D.L. (2004). Audism: Exploring the Metaphysics of Oppression. J. Deaf Stud. Deaf Educ..

[36] Piketh R.W., Kometsi K. (2025). A Purposive Sampling-Based Narrative Literature Review of Understanding Men’s Help-Seeking Behaviours in Selected Literature. J. Ment. Health Soc. Behav..

[37] Palinkas L.A., Horwitz S.M., Green C.A., Wisdom J.P., Duan N., Hoagwood K. (2015). Purposeful sampling for qualitative data collection and analysis in mixed method implementation research. Adm. Policy Ment. Health Ment. Health Serv. Res..

[38] Motevalli M. (2025). Comparative analysis of systematic, scoping, umbrella, and narrative reviews in clinical research: Critical considerations and future directions. Int. J. Clin. Pract..

[39] Sukhera J. (2022). Narrative reviews: Flexible, rigorous, and practical. J. Grad. Med. Educ..

[40] Ervin A., Raphael D. (2026). Liberal/individualized versus materialist/structuralist approaches to addressing social and health inequalities: Education and income as social determinants of health. Community Health Equity Res. Policy.

[41] Poirier B., Haag D., Soares G., Jamieson L. (2023). Whose values, what bias, which subjectivity?: The need for reflexivity and positionality in epidemiological health equity scholarship. Aust. N. Z. J. Public Health.

[42] The Daily Moth (2025). Cuts to NIH Funding Have Dismantled a Vital “Deaf Scientists Pipeline,” Affecting Deaf Researchers [Video]. YouTube. https://www.youtube.com/watch?v=3i0Q5OLZ54U.

[43] Sarkis-Onofre R., Catalá-López F., Aromataris E., Lockwood C. (2021). How to properly use the PRISMA Statement. Syst. Rev..

[44] Ferrari R. (2015). Writing narrative style literature reviews. Med. Writ..

[45] Rother E.T. (2007). Systematic literature review x narrative review. Acta Paul. Enferm..

[46] Sequera M., Singh S., Fernandes L., Gaikwad L., Gupta D., Chibanda D., Nadkarni A. (2022). Adolescent Health Series: The status of adolescent mental health research, practice, and policy in sub-Saharan Africa: A narrative review. Trop. Med. Int. Health.

[47] Pitchika V., Buttner M., Schwendicke F. (2024). Artifical intelligence and personalized diagnostics in periodontology: A narrative review. Periodontology.

[48] Ignatowicz A., Tarrant C., Mannion R., El-Sawy D., Conroy S., Lasserson D. (2023). Organizational resilience in healthcare: A review and descriptive narrative synthesis of approaches to resilience measurement and assessment in empirical studies. BMC Health Serv. Res..

[49] Zahid M.J., Mavani P., Awuah W.A., Alabdulrahman M., Punukollu R., Kundu A., Mago A., Maher K., Adebusoye F.T., Khan T.N. (2024). Sculpting the future: A narrative review of 3D printing in plastic surgery and prosthetic devices. Health Sci. Rep..

[50] Ghaempanah F., Moasses Ghafari B., Hesami D., Hossein Zadeh R., Noroozpoor R., Moodi Ghalibaf A., Hasanabadi P. (2024). Metaverse and its impact on medical education and health care system: A narrative review. Health Sci. Rep..

[51] Spoelder E.J., Slagt C., Scheffer G.J., Van Geffen G.J. (2022). Transport of the patient with trauma: A narrative review. Anaesthesia.

[52] Halloy A., Simon E., Hejoaka F. (2023). Defining patient’s experiential knowledge: Who, what and how patients know. A narrative critical review. Sociol. Health Illn..

[53] Braun V., Clarke V. (2023). Toward good practice in thematic analysis: Avoiding common problems and be(com)ing a knowing researcher. Int. J. Transgender Health.

[54] Ho L., Limpaecher A. (2022). The Importance of Reflexivity in Qualitative Research. Essential Guide to Coding Qualitative Data.

[55] Braun V., Clarke V., Cooper H., Coutanche M.N., McMullen L.M., Panter A.T., Rind-Skopf D., Sher K.J. (2023). Thematic analysis. APA Handbook of Research Methods in Psychology: Research Designs: Quantitative, Qualitative, Neuropsychological, and Biological.

[56] Schniedewind E., Lindsay R., Snow S. (2020). Ask and ye shall not receive: Interpreter-related access barriers reported by Deaf users of American sign language. Disabil. Health J..

[57] Panko T.L., Cuculick J., Albert S., Smith L.D., Cooley M.M., Herschel M., Mitra M., McKee M. (2023). Experiences of pregnancy and perinatal healthcare access of women who are deaf: A qualitative study. BJOG Int. J. Obstet. Gynaecol..

[58] Mussallem A., Panko T.L., Contreras J.M., Plegue M.A., Dannels W.A., Roman G., Hauser P.C., McKee M.M. (2022). Making virtual health care accessible to the deaf community: Findings from the telehealth survey. J. Telemed. Telecare.

[59] James T.G., Coady K.A., Stacciarini J.-M.R., McKee M.M., Phillips D.G., Maruca D., Cheong J. (2022). “They’re Not Willing To Accommodate Deaf patients”: Communication Experiences of Deaf American Sign Language Users in the Emergency Department. Qual. Health Res..

[60] National Association of the Deaf (2023). Real Talk, Good Action–Healthcare System: The Bad and the Ugly [Video]. YouTube. https://www.youtube.com/watch?v=hcPrvoNJo9I.

[61] Michigan Medicine (2022). Racism and Audism: Their Impact on Black Deaf Health [Video]. YouTube. https://www.youtube.com/watch?v=uK9M5Fd0V6A.

[62] NHS Deaf Mental Health Working Group (2023). Shaping the Future of Deaf Mental Health. https://signhealth.org.uk/wp-content/uploads/2023/07/Report-Shaping-the-future-of-deaf-mental-health.pdf.

[63] Lim T., Tsai M., Reyes A., Kapanen A., D’Amato F. (2025). Deaf, deaf-blind, and hard of hearing needs and perceptions of community pharmacy services. Can. Pharm. J. Rev. Pharm. Can..

[64] Jaiswal A., Minhas R., Madho K., Keyes K., Spruyt-Rocks R. (2020). Service Gaps, Prevalence and Severity Specific Estimates of Deafblindness in Canada: A Report. DBI Rev..

[65] Franklin A. (2021). Rochester’s Black Deaf Community Speaks Up About Barriers to Health Care. WXXI News. https://www.wxxinews.org/inclusion-desk/2021-06-14/rochesters-black-deaf-community-speaks-up-about-barriers-to-health-care.

[66] Fernández-Valderas C., Macías-Seda J., Gil-García E. (2017). Experiences of deafblind people about health care. Enfermería Clínica Engl. Ed..

[67] Blind Citizens Australia (2020). Access to Health Services for People Who Are Blind or Vision Impaired. https://www.bca.org.au/wp-content/uploads/2020/10/Health-Care-Policy-Aug-2020.docx.

[68] Oviedo-Cáceres M.D.P., Arias-Valencia S., Hernández-Quirama A., Ruiz-Rodríguez M., Guisasola-Valencia L. (2023). Intersectionality and access to visual rehabilitation services: Experiences of people with low vision, a qualitative study. Br. J. Vis. Impair..

[69] Conner K.R., Jones C.M., Wood N., Aldalur A., Paracha M., Powell S.J., Nie Y., Dillon K.M., Rotoli J. (2023). Use of Routine Emergency Department Care Practices with Deaf American Sign Language Users. J. Emerg. Med..

[70] Helm K.V.T., Panko T.L., Herschel M., Smith L.D., Mitra M., McKee M.M. (2023). Maternal Health Experiences of Black Deaf and Hard of Hearing Women in the United States. Women’s Health Issues.

[71] Brunes A., Hansen B., Heir M. (2019). Loneliness among adults with visual impairment: Prevalence, associated factors, and relationship to life satisfaction. Health Qual. Life Outcomes.

[72] McKee M.M., Winters P.C., Sen A., Zazove P., Fiscella K. (2015). Emergency Department utilization among Deaf American Sign Language users. Disabil. Health J..

[73] Paracha M., Wagner E., Brumfield O., Winninghoff J., Wright J., Rotoli J., Hauser P. (2023). Medication-Related Experience of Deaf American Sign Language Users. HLRP Health Lit. Res. Pract..

[74] CHS Canada TV (2013). CHS Position Paper-Challenges Affecting the Deaf and Interpreter Communities [Video]. YouTube. https://www.youtube.com/watch?v=OuPsJU0KAqg.

[75] Russell D., Chovaz C., Boudreault P. (2018). Administration of Justice: The Experiences of Deaf, DeafBlind, and Deaf People with Additional Disabilities in Accessing the Justice System.

[76] Sharts-Hopko N.C., Smeltzer S., Ott B.B., Zimmerman V., Duffin J. (2010). Healthcare Experiences of Women With Visual Impairment. Clin. Nurse Spec..

[77] Beverley C.A., Bath P.A., Barber R. (2011). Health and social care information for visually-impaired people. Aslib Proc..

[78] Cupples M.E., Hart P.M., Johnston A., Jackson A.J. (2012). Improving healthcare access for people with visual impairment and blindness. BMJ.

[79] SignHealth (2014). A Report into the Health of Deaf People in the UK. https://signhealth.org.uk/wp-content/uploads/2019/12/THE-HEALTH-OF-DEAF-PEOPLE-IN-THE-UK-.pdf.

[80] Jin E.Y.W., Daly B. (2010). The self-reported oral health status and behaviors of adults who are deaf and blind. Spec. Care Dent..

[81] Buettgen A. (2025). Poverty and Economic Exclusion: A Critical Disability Studies Perspective. Curr. Dev. Disord. Rep..

[82] Harding S. (1992). Rethinking Standpoint Epistemology: What Is “Strong Objectivity?”. Centen. Rev..

[83] Veitch A., Rinaldi J. (2024). Disability Research Principles: Lessons from a Speaker Series. Crit. Stud. Int. Interdiscip. J..

